# Molten Aluminum Ocular Thermal Burn: A Unique Opportunity to Gauge the Impact of Thermal Energy

**DOI:** 10.7759/cureus.81639

**Published:** 2025-04-03

**Authors:** Lik Wei Tiong

**Affiliations:** 1 Ophthalmology, Sarawak General Hospital, Kuching, MYS

**Keywords:** aluminum, burn injury, molten metal, ocular burn, ocular contact burn

## Abstract

Burn injuries can be subdivided into two groups: flame burns and contact burns. Ocular contact burns are more uncommon but tend to lead to more severe ocular injury. This case report describes a Roper-Hall grade 3 ocular thermal injury from molten aluminum and aims to estimate the thermal energy involved to improve future injury assessment. A 32-year-old male Myanmar migrant worker presented with a history of having molten aluminum splashed into his left eye from a workplace accident. A hardened metal cast of approximately 1.5 cm in diameter was observed buried in his inferior fornix. The severity of tissue destruction in ocular thermal injury depends on at least four factors: temperature of the agent, heat-retaining capacity of the material, duration of contact, and area over which the heat is applied. When the source is hot and has significant heat retention capacity, the result may be a severe burn with the involvement of the deeper layers of the cornea. While retrospective analyses of human ocular burns exist, these studies lack the controlled quantification of thermal energy, exposure duration, and affected area needed to establish robust correlations with clinical outcomes. Molten metal and glass ocular burns differ from other contact burns; the retained material allows for direct thermal energy calculation. This case underscores the importance of detailed reporting of the retained material in cases of ocular burns involving molten metal and glass. It could provide thermal energy estimation for the observed clinical presentation and outcome. With enough data, it may be helpful in predicting the prognosis of such cases.

## Introduction

Thermal and chemical ocular burns are medical emergencies that require prompt treatment. The usual procedures of obtaining a full medical history and performing a detailed ophthalmic examination are delayed until after treatment initiation.

Thermal injuries, comprising about 90% of all burns, vary in depth based on heat intensity and exposure time. These injuries include scalds (hot liquid immersion), dry heat burns (flame or radiant heat), and contact burns (direct contact with hot objects) [1]. Approximately 7.5-27% have either ocular or lid involvement [2-4], and 1% have corneal burns [4]. Flame burns are the most common cause [2-4].

Ocular contact burns, although more uncommon, tend to lead to more severe ocular injury. Industrial contact burns frequently result from hot metal or solder, while household burns are more often caused by hot grease or oil [5]. A study by Vajpayee et al. involving 59 corneal contact burns found boiling liquids and hot oil accounted for 42%, with firecrackers and matches causing 18% and 17%, respectively [6]. Although multiple reports can be found regarding corneal contact burns due to curling irons in the 1980s [7-9], Boone et al. reported only one case of contact with hot metal out of 25 cases of thermal burn eye injuries from 1750 burn admissions [4]. As for molten metal ocular thermal injuries, only one reported case of molten bronze, one case of molten silver [10], and one case of molten aluminum [11] could be found at the time of writing. Of these, the solidified cast of the molten metal was recovered from the ocular surface of two out of three of these cases.

Ocular burn severity is typically assessed using the Roper-Hall and Dua classifications. The Roper-Hall classification primarily assesses corneal appearance and limbal ischemia [12], while the Dua classification offers a more comprehensive approach, incorporating a detailed assessment of limbal involvement (quantified using a clock-hour system) and the percentage of conjunctival surface area affected [13].

This case report presents a patient with Roper-Hall grade 3 ocular surface and grade 2 eyelid burns resulting from molten aluminum exposure. By estimating the thermal energy delivered using the retained metal, we aim to contribute to a more accurate understanding of injury severity in contact ocular burns.

## Case presentation

**Figure 1:**
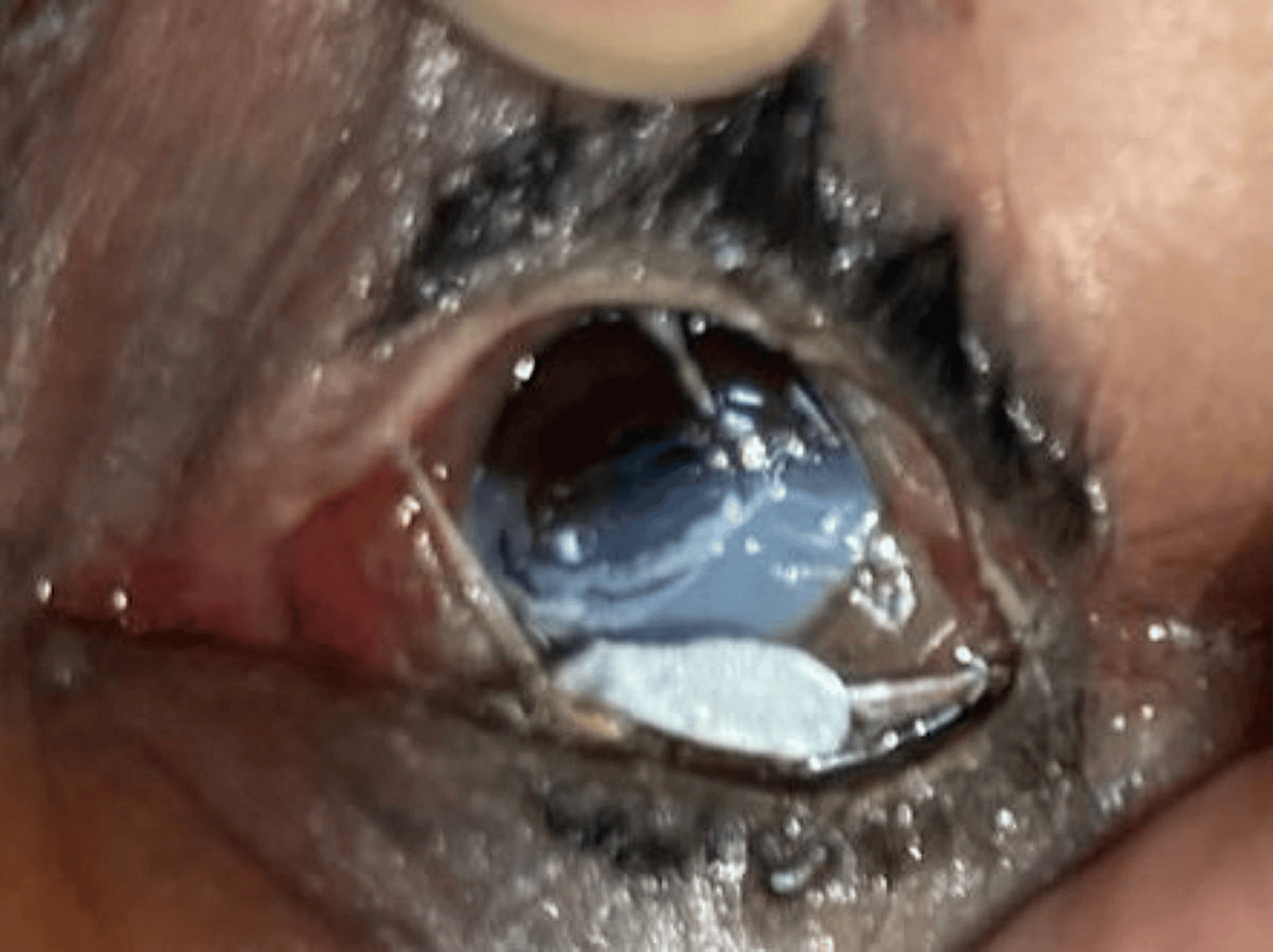
On day one, the patient presents with an aluminum cast retained in the inferior fornix

A 32-year-old male Myanmar migrant worker presented with left eye (LE) blurring of vision and pain after molten aluminum splashed into his LE from a workplace accident. He is unsure whether the splash was from an air pocket rising to the surface or an object descending into the vat, but he was not wearing his face shield at the time. He immediately irrigated his eye under a running tap for 10 minutes prior to presenting to the emergency department. A hardened aluminum casting, approximately 1.5 cm in diameter, was found in the inferior fornix during ocular irrigation (Figure 1).

The vision on presentation was right eye (RE) 6/6, LE hand movement (HM), and no relative afferent pupillary defect (RAPD). There were superficial second-degree burns overlying the left upper and lower eyelids, measuring 2x2 cm. His lashes were singed, the lid was swollen, the conjunctiva was injected and chemosed, and the inferior half of the cornea that was in contact with the aluminum was flattened and opaque. There was an epithelial defect over 90% of the cornea and limbal ischemia from 4-8 o'clock. There was no traumatic cataract or phacodonesis. Intraocular pressure (IOP) was 10;10. The fundoscopic examination revealed a flat, unremarkable posterior segment with no evidence of retinal breaks, detachment, choroiditis, or retinitis.

**Figure 2:**
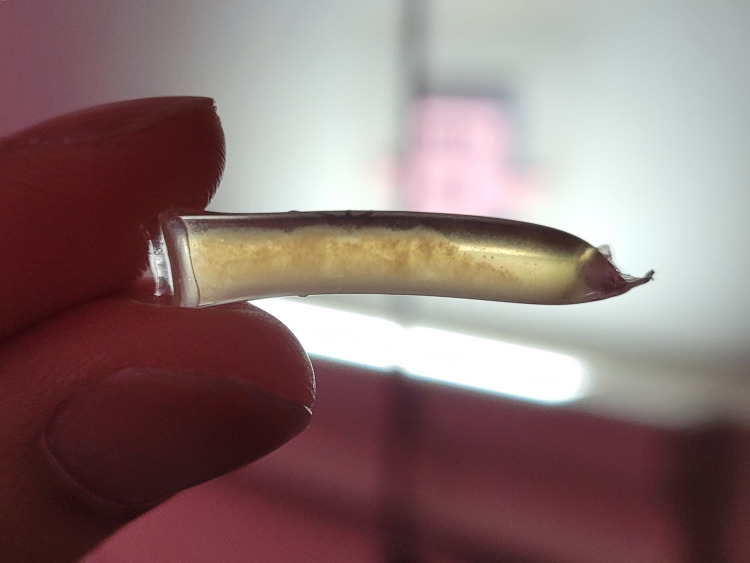
Minim of autologous serum

According to the Roper-Hall classification [12], based on the 1/3 limbal ischemia, our patient had a grade 3 burn with a guarded prognosis. Under the Dua classification, 4 o'clock hours of limbal involvement is a grade 3; bulbar conjunctiva involvement of 25% is grade 2, which is a good prognosis [13]. Gutt moxifloxacin every four hours LE, Gutt dexamethasone every four hours LE, Gutt preservative-free artificial tears every four hours LE, Tab doxycycline 100 mg BD, Tab vitamin C 1 g OD, Occ chloramphenicol QID LA to lids, Gutt autologous serum every two hours LE (Figure 2) was started.

**Figure 3:**
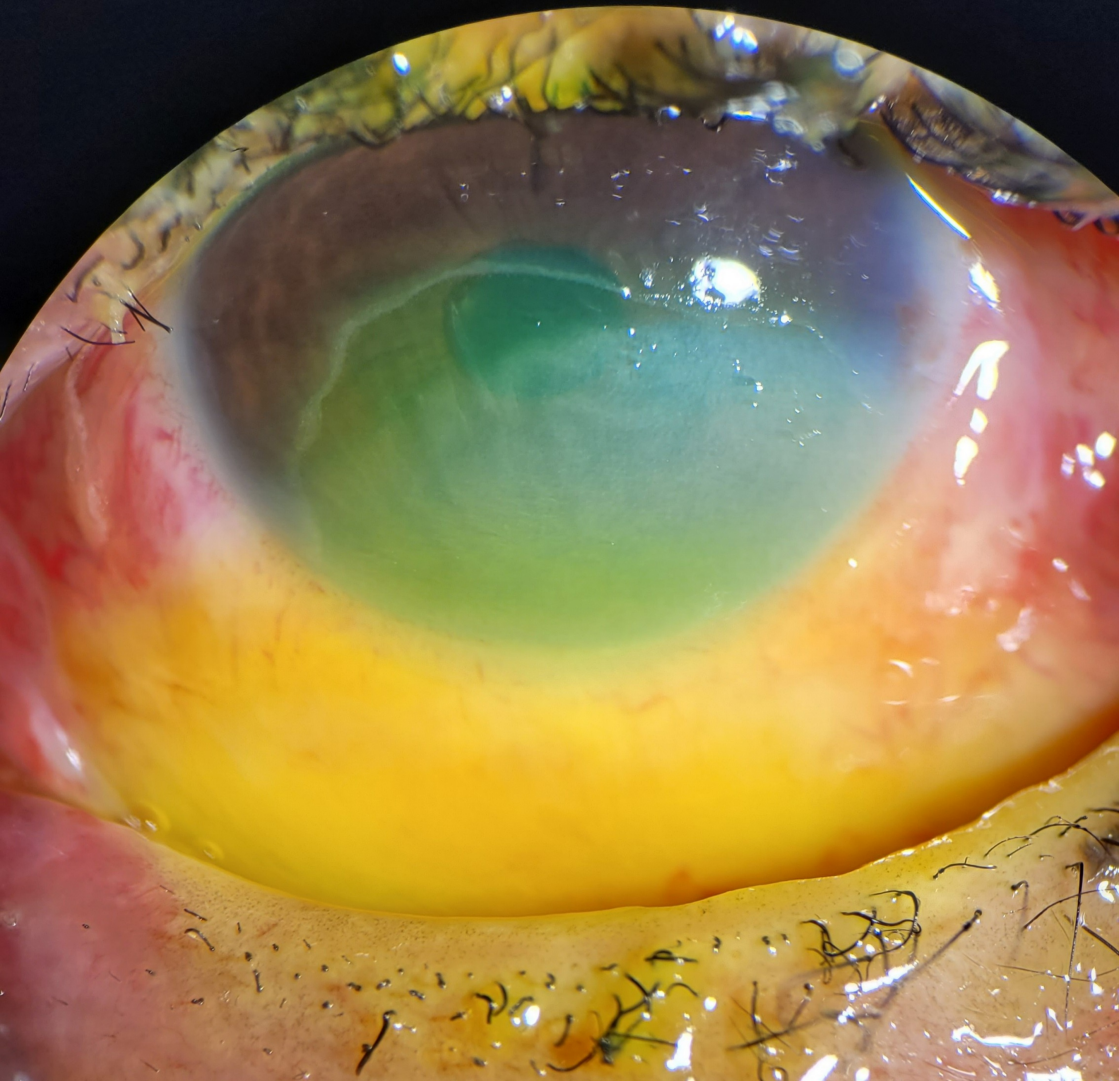
Day seven anterior segment photo, showing the persistent epithelial defect, stroma opacity, 1/3 limbal ischemia, and 25% bulbar conjunctiva involvement

**Figure 4:**
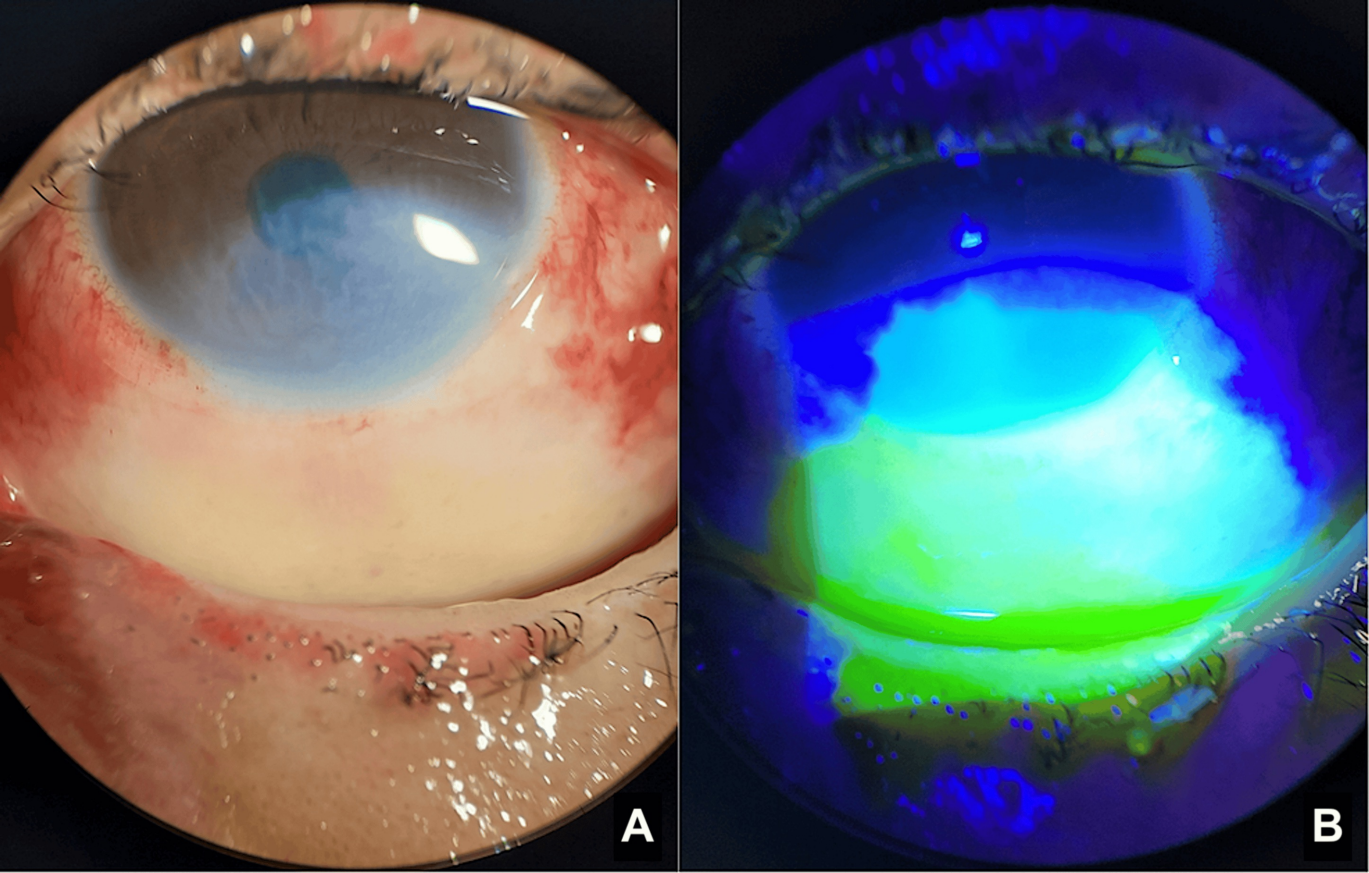
Day 14: Anterior segment photos without filter (A) and with cobalt blue filter and fluorescein stain (B) showing improved corneal stromal opacity and epithelial defect

**Figure 5:**
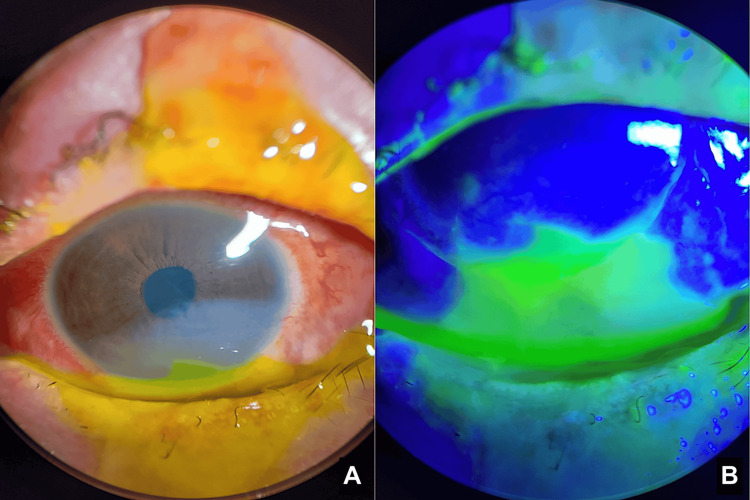
Day 21: Anterior segment photos showing reduced corneal opacity (A) and near-complete re-epithelialization of the corneal epithelial defect and improving bulbar conjunctival defect (B; cobalt blue filter and fluorescein stain)

During the first two days, the corneal epithelial defect rapidly recovered in the areas unaffected by limbal ischemia. The number of clock hours of limbal ischemia, corneal haze, and epithelial defect slowly improved over the next three weeks (Figures 3-5).

## Discussion

The severity of tissue damage from thermal eye injuries is a function of the temperature of the burning agent, its ability to retain heat, the duration of contact, and the size of the affected area [14]. The effects of high temperatures on the ocular surface have been the subject of several studies [15-17]. Goldblatt et al. observed diffuse corneal stromal edema and endothelial cell loss in rabbit corneas following exposure to 59°C for 15 minutes via a heat conductor. Their findings demonstrated that even moderate corneal heating over extended periods causes substantial tissue damage [16].

There is no human equivalent of Goldblatt et al.'s work, and it does not account for higher temperatures and deeper ocular structures such as the sclera, ciliary body, and chorioretina. Cases of molten metal and glass ocular burns provide a unique opportunity. Often, the offending substance can be found on the ocular surfaces of these cases. With known melting points, mass, and contact surface, it provides insight into the amount of thermal energy inflicted onto the eye's surface.

**Figure 6:**
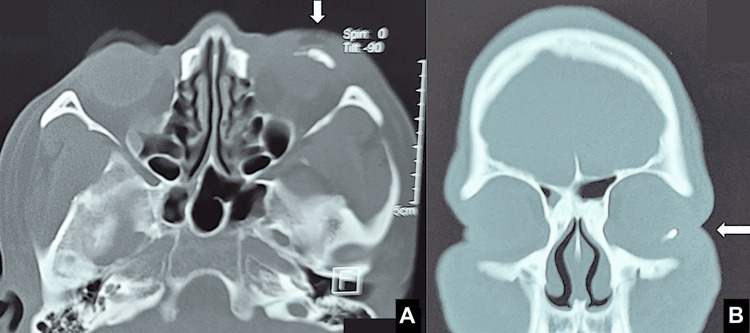
Axial (A) and coronal (B) CT images of the head and orbits. Arrows indicate the molten aluminum cast on the left eye

The melting temperature of pure aluminum is 660.32°C, with a density of 2.70 g/cm^3^ and a heat capacity of 0.9 J/g [18]. The molten metal burnt our patient's eyelid, and a small amount of it settled within his LE inferior fornix, with the resulting ingot forming a cast on the anterior surface of the eye [5] before being removed around eight hours post-trauma. The increased duration of heat conduction may have contributed to the severity of the heat-related damage. The metal would have cooled from above its melting point of 660.32°C to the body temperature of 37.3°C. Unfortunately, this cast had been discarded prior to the acquisition of a photograph, measurements of the contact surface area, volume assessment with a displacement can, and weight determination. However, based on the dimensions from the CT scan (Figure 6), the aluminum cast measures 0.9x1.6x0.6 cm, and an estimated mass of 2.333g can be derived based on the elemental density.

Thermal energy transfer (Q) can be calculated using the formula $Q=m&sdot;c&sdot;&Delta;T$, where Q = thermal energy transferred in joules (J); m = mass of the material in kilograms (kg); c = specific heat capacity of the material in J/kg; and ΔT = change in temperature in Celsius (°C). So in our case, $Q = 2.333g &times; 0.9 J/g &times; (660.32&deg;C - 37.3&deg;C) = 1308.155 J$.

With half the surface in contact with the globe and the other half with the lid and air, if the surface in contact with the globe is estimated to be 1.44 cm^2^, approximately 454.221 J/cm^2^ could have been delivered to the globe surface. To put this in perspective, 4.184 J is needed to raise each mL of water by 1°C at sea level; 1308.155 J is enough to bring 4.984 mL of water to a boil from 37.3°C, or 14.407 mL of water from 37.3°C to 58.9°C. The human eye only has an average volume of 6.5 mL [19].

This estimation of thermal energy is subject to limitations. The purity of the molten aluminum was assumed. Its temperature was assumed to be at melting and not above. The mass of the molten aluminum was estimated using CT scan measurements, introducing uncertainty due to the irregular shape. Furthermore, the calculation assumes uniform heat distribution, a simplification given the likely non-uniform heat transfer across the contact area. These factors could lead to an overestimation or underestimation of the actual thermal energy delivered to the ocular tissues.

Additionally, not all the estimated thermal energy is delivered to surrounding ocular tissue due to the instantaneous formation of a protective film of vapor when the hot metal comes in contact with the tear film [5]. However, when the source is hot and has significant heat retention capacity, such as molten metal or glass, this protective effect is often insufficient, resulting in severe burns involving deeper layers [20].

Current grading systems are mainly concerned about the extent of ocular surface involvement and limbal involvement [12,13], but not the depth of injury and the deeper underlying structures. Quantifying the amount of heat energy in joules and its concentration on the contact surface in J/cm^2^ provides an objective numerical value for the severity of the clinical picture of contact burn. Threshold values for when the tear film is no longer sufficient to protect the ocular surface, as well as values when hypotony due to cyclodestruction, globe perforation, or phthisis bulbi occurs, may be identified.

Sufficient data correlating these values to the severity and outcome of the burns may assist in prognostic predictions for contact burns. Unfortunately, at this time, only three reported cases can be found in the literature, and neither of the two cases where metal casts were recovered from the ocular surface provided sufficient details regarding the mass or volume of the recovered metal cast.

## Conclusions

Molten metal and glass ocular burns are a severe subset of contact ocular burns, as the offending substance can often be found on the ocular surface. With known melting points, mass, and contact surface, it provides insights into the amount of thermal energy that led to the extent of injury observed. However, accurate thermal energy estimation requires improved data collection, as demonstrated by the limitations of the current case. The collection of further cases, along with accurate thermal energy calculations and correlation with injury severity and outcome, will be crucial for developing a more robust predictive model for prognosis and complications in such cases.
